# To the Editors of the Medical and Physical Journal

**Published:** 1801-09

**Authors:** W. B. Bayley

**Affiliations:** North Allerton


### *To the Editors of the Medical and Phyfical Journal.*

GENTLEMEN,

ON perufing your ufeful Journal for laft month, I obferved two cafes of Jaundice very accurately defcribed by Dr. Crichton, proceeding from a caufe, as the Doctor fuppofed, hitherto undefcribed.

It is very improbable that fuch an infignificant publication as my Thefis, (De Ictero,) publifhed in the year 1799, fhould ever fall into the Doctor's hands, I therefore take the liberty of mentioning that I there gave, as one of the remote caufes of jaundice, a fchirrus of the pancreas, and had the fatisfaction of having my opinion *fully* corroborated by a preparation of that organ, in a fchirrous ftate, being fhewn by Dr. Monro of Edinburgh, at his lecture, and which had been the caufe of a jaundice of two years ftanding, as appeared upon diffection.

I am fure I need not make any apology to Dr. Crichton for the above ftatement, being well convinced that he will receive fatisfaction from any further teftimony in favor of a medical fact he has fo correctly given to the world. I am, &c.

